# A mutation in mannose‐phosphate‐dolichol utilization defect 1 reveals clinical symptoms of congenital disorders of glycosylation type I and dystroglycanopathy

**DOI:** 10.1002/jmd2.12060

**Published:** 2019-09-30

**Authors:** Walinka van Tol, Angel Ashikov, Eckhard Korsch, Nurulamin Abu Bakar, Michèl A. Willemsen, Christian Thiel, Dirk J. Lefeber

**Affiliations:** ^1^ Department of Neurology, Donders Institute for Brain, Cognition and Behavior Radboud University Medical Center Nijmegen The Netherlands; ^2^ Translational Metabolic Laboratory, Department of Laboratory Medicine, Radboud Institute for Molecular Life Sciences Radboud University Medical Center Nijmegen The Netherlands; ^3^ Children's Hospital of the City of Cologne Cologne Germany; ^4^ Department of Pediatric Neurology, Amalia Children's Hospital, Donders Institute for Brain, Cognition and Behavior Radboud University Medical Center Nijmegen The Netherlands; ^5^ Center for Child and Adolescent Medicine, Kinderheilkunde I University of Heidelberg Heidelberg Germany

**Keywords:** congenital disorders of glycosylation, dolichol‐phosphate‐mannose, dystroglycanopathy, MPDU1‐CDG

## Abstract

Congenital disorders of glycosylation type I (CDG‐I) are inborn errors of metabolism, generally characterized by multisystem clinical manifestations, including developmental delay, hepatopathy, hypotonia, and skin, skeletal, and neurological abnormalities. Among others, dolichol‐phosphate‐mannose (DPM) is the mannose donor for N‐glycosylation as well as O‐mannosylation. DOLK‐CDG, DPM1‐CDG, DPM2‐CDG, and DPM3‐CDG are defects in the DPM synthesis showing both CDG‐I abnormalities and reduced O‐mannosylation of alpha‐dystroglycan (αDG), which leads to muscular dystrophy‐dystroglycanopathy. Mannose‐phosphate‐dolichol utilization defect 1 (MPDU1) plays a role in the utilization of DPM. Here, we report two MPDU1‐CDG patients without skin involvement, but with massive dilatation of the biliary duct system and dystroglycanopathy characteristics including hypotonia, elevated creatine kinase, dilated cardiomyopathy, buphthalmos, and congenital glaucoma. Biochemical analyses revealed elevated disialotransferrin in serum, and analyses in fibroblasts showed shortened lipid linked oligosaccharides and DPM, and reduced O‐mannosylation of αDG. Thus, MPDU1‐CDG can be added to the list of disorders with overlapping biochemical and clinical abnormalities of CDG‐I and dystroglycanopathy.

**Synopsis:**

Mannose‐phosphate‐dolichol utilization defect 1 patients can have overlapping biochemical and clinical abnormalities of congenital disorders of glycosylation type I and dystroglycanopathy.

## 1 INTRODUCTION

The congenital disorders of glycosylation (CDG) are inborn errors of metabolism with a great genetic heterogeneity. CDG‐I defects are located in the assembly of the lipid linked oligosaccharide (LLO) glucose_3_mannose_9_ *N*‐acetylglucosamine_2_‐PP‐dolichol (Glc_3_Man_9_GlcNAc_2_‐PP‐Dol) or the transfer of its oligosaccharide to proteins in the endoplasmic reticulum (ER). CDG‐I leads to multiorgan phenotypes, including disorders of the brain and neuromuscular system, hepatopathy, skin, and skeletal abnormalities.^1^

Genetic defects in the biosynthesis of dolichol‐phosphate‐mannose (DPM; NUS1‐CDG, DHDDS‐CDG, PMM2‐CDG, SRD5A3‐CDG, DOLK‐CDG, DPM1‐CDG, DPM2‐CDG, DPM3‐CDG) lead to CDG‐I profiles of transferrin. However, the clinical phenotypes associated with these defects are very different, which is partly explained by that DPM is required for N‐glycosylation, O‐mannosylation, C‐mannosylation, and GPI‐anchor synthesis. In DOLK‐CDG, DPM1‐CDG, DPM2‐CDG, and DPM3‐CDG, biochemical and phenotypic abnormalities overlap with CDG‐I and muscular dystrophy‐dystroglycanopathy, which is caused by reduced O‐mannosylation of alpha‐dystroglycan (αDG).^2^, ^3^, ^4^, ^5^

Mannose‐phosphate‐dolichol utilization defect 1 (MPDU1) is involved in the flipping of DPM and dolichol‐phosphate‐glucose (DPG) across the ER membrane and for efficient use of DPM and DPG within the ER lumen.^6^, ^7^ MPDU1‐CDG patients have CDG‐I with epilepsy, psychomotor retardation, and skin abnormalities.^7^, ^8^, ^9^ Here, we describe two siblings with a G73E substitution in MPDU1 without skin involvement, but with dilatation of the biliary ducts and dystroglycanopathy symptoms including elevated creatine kinase (CK), dilated cardiomyopathy (DCM), buphthalmos, and glaucoma.

## 2 MATERIALS AND METHODS

### 2.1 Subjects

Plasma and fibroblasts were obtained for CDG diagnostics in the Radboudumc Expertise Center for Disorders of Glycosylation in accordance with the Declaration of Helsinki. Informed consent was obtained from patients or their legal representatives. Written informed consent was obtained for inclusion of facial images.

### 2.2 CDG diagnostics

Serum transferrin isoelectric focusing (TIEF) and electrospray ionization mass spectrometry (ESI‐MS) were performed as described.^10^, ^11^, ^12^ Whole exome sequencing (WES), high‐performance liquid chromatography (HPLC), and thin‐layer chromatography (TLC) of LLOs and DPM were performed as described elsewhere.^13^, ^14^

### 2.3 Immunoblotting and laminin overlay assay

Fibroblasts were cultured in Dulbecco's Modified Eagle Medium (DMEM) with 10% fetal calf serum (Gibco) and 1% penicillin‐streptomycin (Gibco). Fibroblast lysates were enriched for glycoproteins using agarose‐conjugated wheat germ agglutinin (Sigma). Proteins were loaded on 10% polyacrylamide gels and transferred to nitrocellulose membranes by western blotting. Membranes were used for laminin overlay (LO)^4^, ^15^ or incubated with primary antibodies against glycosylated αDG (IIH6C4, 1:2500, Merck 05‐593), the dystroglycan core‐protein (DAG1, 1:333, Genetex), or βDG (1:250, Novacastra) and with HRP‐conjugated polyclonal goat anti‐rabbit or goat anti‐mouse antibodies (1:5000, DAKO).

## 3 RESULTS

### 3.1 Clinical description

**Figure 1:**
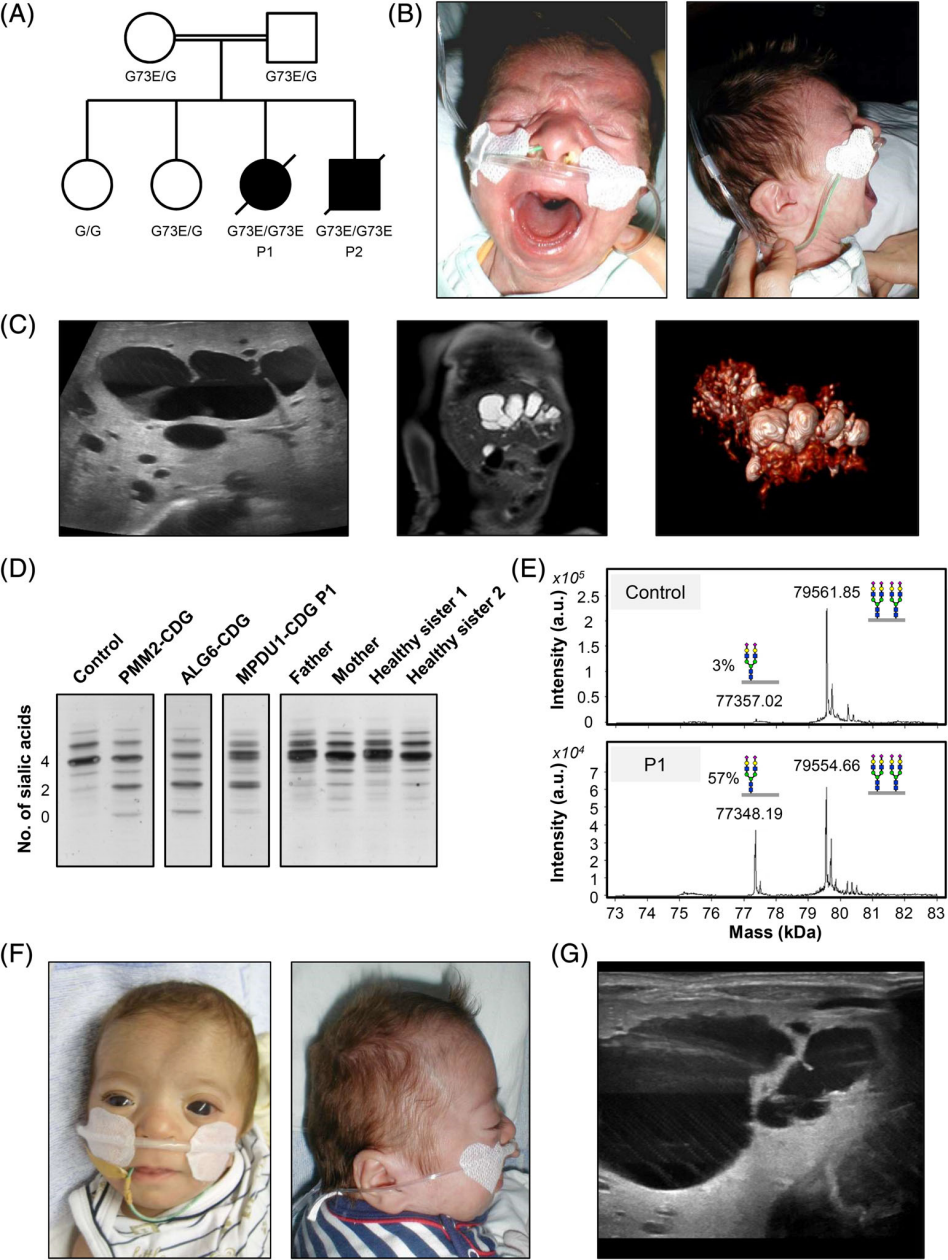
Family pedigree and clinical images of affected patients. A, Family pedigree of MPDU1 patients (P1 = patient 1, P2 = patient 2). B, Front and side facial view of patient 1. C, Abdominal sonography (left), magnetic resonance imaging (middle), and 3D image from magnetic resonance cholangiopancreatography (right) of patient 1 showing a vast dilatation of the complete intrahepatic biliary duct system. D, Serum transferrin isoelectric focusing (TIEF) analysis of P1, her parents and two healthy sisters. E, ESI‐MS of serum transferrin. Disialotransferrin levels are expressed as a percentage of tetrasialotransferrin. F, Front and side facial view of patient 2 at 4 months of age. G, Abdominal sonography of patient 2, showing dilatation of the intrahepatic biliary duct. CDG, congenital disorders of glycosylation; ESI‐MS, electrospray ionization mass spectrometry; MPDU1, mannose‐phosphate‐dolichol utilization defect 1

The two patients are brother and sister from Iraqi origin. They were the third and fourth child of consanguineous parents (first cousins), and had two healthy sisters (Figure 1A). Table 1 summarizes the clinical features of these two patients and four previously reported MPDU1‐CDG patients.^7^, ^8^, ^9^

**Table 1:** Clinical and laboratory data of the two presented patients and the patients from the literature

Patient	Patient 1	Patient 2	Patient girl	Patient S	Patient L	Patient A	Patient boy
Described in	This paper	This paper	Thiel et al^9^	Schenk et al^7^	Schenk et al^7^	Schenk et al^7^	Kranz et al^8^
Sex	Female	Male	Female	Male	Female	Male	Male
Zygosity	Homozygous	Homozygous	Homozygous	Homozygous	Compound heterozygous	Homozygous	Homozygous
Nucleotide change							
Chr17[GRCh38]	g.7585994G>A	g.7585994G>A	g.7585994G>A	g.7585994G>A	g.7583864T>C	g.7586745T>C	g.7585997T>C
NM_004870.3	c.218G>A	c.218G>A	c.218G>A	c.218G>A	c.2T>C g.7587164del c.511delC	c.356T>C	c.221T>C
Protein change	p.G73E	p.G73E	p.G73E	p.G73E	p.M1T (loss of start codon) p.L171Sfs*42	p.L119P	p.L74S
Affected exon(s)	3	3	3	3	1 and 6	4	3
Parental consanguinity	+	+	+	+	No	+	No
Family	Two healthy older sisters	Brother of patient 1, two healthy older sisters	Healthy older sister and twin brother, older brother showed similar disease and died in the neonatal period	Normal	Brother died at 2 months with similar disease	Normal	n.a.
Pregnancy (weeks)	36 + 2 weeks	30 + 4 weeks placental hypertrophy	30 + 5 weeks	37 weeks	40 weeks	40 weeks	39 weeks
Birth weight (g)	2390	1420	2370	2485	3200	3200	2770
Perinatal problems	Apneas and bradycardias, respiratory failure	Cyanotic, breathless, hypotonia, respiratory failure	Hypotonia, insufficient breathing	Hypotonia, seizures	Hypertonia	No	Hypotonia
Dysmorphology	Smooth philtrum, retrognathia, low‐set, posterior‐rotated ears, hypertelorism	Smooth philtrum, retrognathia, low‐set, posterior‐rotated ears, hypertelorism, micropenis	Hypertelorism, broad‐based nose, thin lips	Large anterior fontanel, bilateral parietal bossing, thin lips	No	No	Contractures
Psychomotor development	Absent	Absent	Absent	Absent	Severe retardation	Severe retardation	Ataxia, profound psychomotor retardation, unable to communicate
Feeding problems	Dysphagia	Dysphagia	Dysphagia	From 4 months	No	No	Deceased food intake, abdominal pain, and frequent vomiting
Hypotonia	+	+	+	+++	No	+	+
Seizures	Seizures with apneas	Seizures with apneas, hypertonic attacks	No	Severe, with apnea	Hypertonic attacks in infancy	Generalized febrile seizures at 15 months	Seizures at 5 months
Electroencephalogram	Multifocal sharp waves	Parieto‐temporo‐occipital and multiregional spikes	Generalized background slowing in the theta and delta frequency ranges but no epileptiform activity	Hypsarrhythmia	Abnormal β‐activity	Generalized dysrhythmia	Hypersynchronic activity
Magnetic resonance imaging of the head	Enlarged subarachnoid space	n.a.	No abnormalities	Normal myelination, enlarged subarachnoid space, enlarged ventricles	Normal myelination	Normal myelination, enlarged frontal spaces	General cerebral atrophy
Tendon reflexes	Normal	Normal	n.a.	Normal	Normal	Normal	Normal
Nerve conduction velocity	n.a.	n.a.	n.a.	Normal	Normal	Normal	n.a.
Ophthalmoscopy	Buphthalmos with congenital glaucoma, pupillary iris pigment epithelial cysts	Buphthalmos with severe congenital glaucoma	n.a.	Optic atrophy	Pale papillae	Normal	Nystagmus, amaurosis, strabismus morphology of the retina normal
Visual and acoustic responses	BERA: pathological	BERA: pathological	n.a.	Absent	Normal	Normal	Amaurosis, BERA normal
Skin disorder	No	No	Small hands with scleroderma‐like consistency and loss of dermatoglyphic patterns, ichthyosis	Deneralized patchy desquamation	Ichthyosis	Transient eczema	Dry, transient hyperkeratotic and scaling with erythroderma
Somatic development	At 4 months	At 9 months	n.a.	At 4 months	At 16 years	At 10 years	Dystrophy with W, H, and HC below P3
	W: 4 kg	W: 5.6 kg		W: P3–1.1 kg	W: P3	W: P97	
				HC: P3–2 cm	H: P3–16 cm	H: P10–25	
					HC: P3 normal pubertal development	HC: P75–97	
Other clinical features	Apneas with desaturations, respiratory insufficiency, death at 4 months	Apneas with desaturations, respiratory insufficiency, death at 11 months	>1.5 Months: microcephaly. Apneas, cyanosis, oxygen dependency, death at 6 months	No weight gain, oxygen dependency, ascites, death at 10 months	n.a.	n.a.	n.a.
Other organ features	Enhanced echogenicity of the marrow pyramids, small renal cysts Massive dilatation of the biliary duct system Postpartual pulmonary hypertension, later dilated cardiomyopathy	Enhanced echogenicity of the marrow pyramids, small renal cysts Massive dilatation of the biliary duct system Dilated aorta ascendens, hypertrophic cardiomyopathy, arterial hypertension	Initial electrocardiogram (ECG): Moderate focal hypertrophy of the basal interventricular septum but no cardiac malformations. ECG at 3 months: severe noncompaction cardiomyopathy	Mild pericardial effusion bilateral small cortical renal cysts	n.a.	n.a.	n.a.
Serum transaminases	Normal	Normal	n.a.	Normal	Normal	Normal	Normal
Other laboratory abnormalities	Thrombocytopenia CK elevated, fibrinogen low, ATIII not measurable	Thrombocytopenia CK elevated, ATIII low	CK elevated	Mild persistent thrombocytopenia, periodic elevation of CK	Transient deficiency of growth hormone and IGF‐I	No	Slightly reduced ATIII

Abbreviations: ATIII, antithrombin III; BERA, brainstem evoked response audiometry; CK, creatine kinase; IGF‐I, insulin‐like growth factor 1; n.a., not available.

#### 3.1.1 Patient 1

Patient P1, a girl, was born spontaneously in the 37th gestational week after an uneventful pregnancy. At birth, her length was 43 cm (standard deviation score, (SDS), −2.08), weight 2390 g (SDS, −0.81), and head circumference 32 cm. Because of postnatal apneas and bradycardias with deep desaturations, followed by respiratory failure, she was resuscitated and referred to the neonatal intensive care unit. Slight dysmorphic features were noted including a smooth philtrum, retrognathia, low‐set, posterior‐rotated ears, and hypertelorism with megalocorneae (Figure 1B).

At the age of 2 months, P1 showed tonic‐clonic seizures with multifocal sharp waves on electroencephalography (EEG). Brain magnetic resonance imaging (MRI) revealed enlarged outer fronto‐temporo‐parietal cerebrospinal fluid spaces. Abdominal sonography as well as magnetic resonance cholangiopancreatography showed a vast dilatation of the intrahepatic biliary ducts, especially within the left lobe of the liver (Figure 1C). There were no further signs of biliary duct obstruction or liver enlargement. Initially, kidney sonography showed enhanced echogenicity of the marrow pyramids without corresponding MRI abnormalities. However, at the age of 7 weeks, small renal cysts were suspected in an ultrasound scan.

Initially, electrocardiography and echocardiography were normal, except for a mild temporary pulmonary hypertension. At the age of 3 months, the patient evolved DCM with low output and a shortening fraction of at least 9%.

The megalocorneae possessed a diameter of 13 mm (normal in newborn = 9.5 mm). Further ophthalmologic examination revealed a buphthalmos with congenital glaucoma. The eye lenses were slightly cloudy and pupillary iris showed pigment epithelial cysts. A sensorineural hearing loss was identified with brainstem evoked response audiometry (BERA) evaluation. There were no abnormalities of the skin.

Blood CK levels were elevated up to 3090 U/L without substantial elevation of aspartate amino transferases (maximal 175 U/L) and alanine amino transferases (maximal 163 U/L), biochemical signs of cholestasis or icterus. There was a marked congenital thrombocytopenia (minimal 45 000 platelets/μL), a slightly decreased fibrinogen (minimal 96 mg/dL), and a nonmeasurable antithrombin III (ATIII, <20%).

Prometaphase karyotype analysis showed a normal 46,XX female karyotype and a normal array‐comparative genomic hybridization (array‐CGH), without any microdeletion or duplication. Congenital toxoplasmosis, other (syphilis, varicella‐zoster, parvovirus B19), rubella, cytomegalovirus, and herpes (TORCH) infections, peroxisomal diseases as well as mucopolysaccharidoses were ruled out by serology and metabolic screening. The serum amino acids, acylcarnitine profile, very long chain fatty acids, 7‐dehydrocholesterol, and urine organic acids were normal. In view of the clinical symptoms of multiorgan involvement with seizures, cardiomyopathy, eye, and liver abnormalities as well as thrombocytopenia and ATIII deficiency, a CDG was suspected and TIEF and ESI‐MS of serum transferrin were performed. These revealed reduced tetrasialotransferrin, with increased asialo‐ and disialotransferrin indicative of a CDG‐I (Figure 1D,E).

In her short life, the patient did not exhibit any psychomotor development, and had to be fed parenterally or by a gastric tube. The clinical course was complicated by an increasing frequency and severity of seizures with epileptic apneas followed by respiratory insufficiency. Congestive heart failure as a consequence of the DCM led to the termination of therapeutic interventions, and she died at the age of nearly 4 months.

#### 3.1.2 Patient 2

This patient exhibited nearly the same clinical features and course as his affected sister (P1). He was born premature per cesarean section in the 31st gestational week because of a rupture of the placental membrane and uterine bleeding. His birth length was 38 cm (SDS, −0.38), his weight was 1420 g (SDS, −0.09), and he had a head circumference of 28 cm. He showed a smooth philtrum, retrognathia, and low‐set, posterior‐rotated ears. He had hypertelorism with prominent appearing eyes with enlarged and cloudy corneae (Figure 1F). Musculature was slightly hypotonic, but otherwise the child appeared to be normal and without skin abnormalities, except a micropenis. After birth, he was cyanotic, breathless, and without muscular tonus, requiring immediate cardiopulmonary resuscitation and controlled ventilation.

Echocardiography detected a dilated aorta ascendens, a transient pulmonary hypertension, and a hypertrophic cardiomyopathy (HCM) at the age of 3 weeks, which developed in combination with an arterial hypertension.

At the age of 6 months, he had generalized seizures accompanied by desaturations, whereas EEG showed parieto‐temporo‐occipital and multiregional spikes over both hemispheres. Sonography of the brain showed no gross abnormalities. Abdominal sonography revealed dilatation of the intrahepatic biliary duct system (Figure 1G). Kidney sonography exhibited enhanced echogenicity of the marrow pyramids. At the age of 4 months, he developed multiple small cysts subcapsular within the renal parenchyma.

The ophthalmological examination revealed a buphthalmos with slightly opaque corneae with a diameter of 11 mm and severe congenital glaucoma, requiring prompt trabeculectomy intervention. The BERA showed a sensorineural hearing loss.

Chromosomal analysis showed a normal 46,XY karyotype, and array‐CGH as well as TORCH serology were normal. He also exhibited thrombocytopenia (minimal 13 000 platelets/μL), elevated CK levels (up to 905 U/L), and low ATIII (maximal 40%), but transaminases, fibrinogen, thyroxin‐binding globulin, and bilirubin were normal.

Like his sister, P2 hardly presented any psychomotor development, and had to be fed parenterally or by a gastric tube. He showed severe apneas and bradycardias which were not treatable, and he died at the age of 11 months from respiratory failure.

### 3.2 Genetic analysis revealed a homozygous missense mutation in MPDU1

Whole exome sequencing revealed a homozygous missense mutation Chr17(GRCh38): g.7585994G>A; NM_004870.3(*MPDU1*): c.218G>A; p.(G73E) in both patients. This mutation has been reported in two other MPDU1‐CDG patients.^7^, ^9^ WES was also performed of the patients' healthy sisters and parents and confirmed parental segregation of the mutation (Figure 1A).

### 3.3 Biochemical analysis revealed elevated LLO intermediates, DPM, and reduced O‐mannosylation of αDG

**Figure 2:**
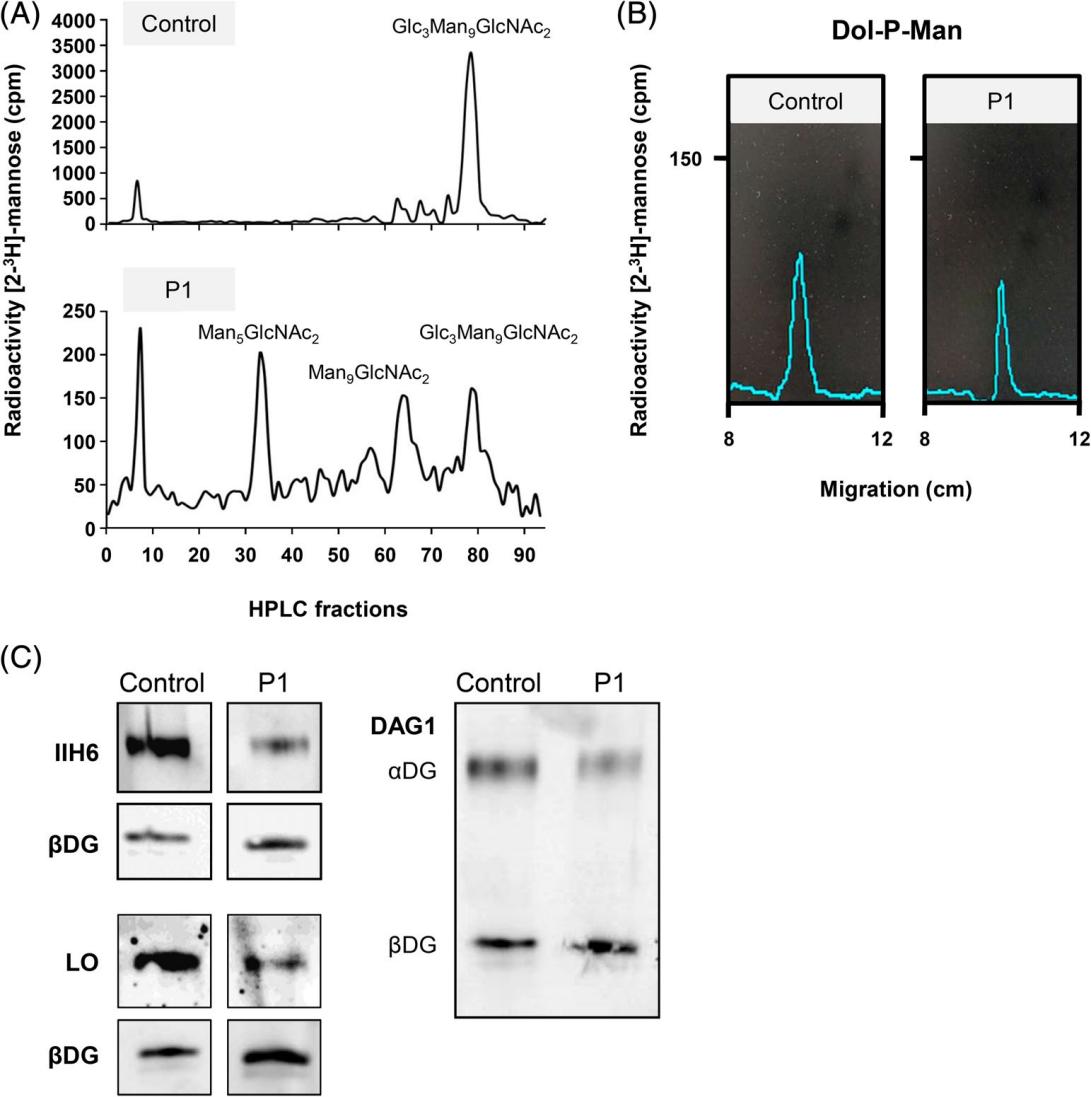
CDG diagnostics and biochemical analyses of MPDU1‐CDG P1. A, HPLC analysis of LLO from fibroblasts of patient 1 (P1) and a control revealed the accumulation of the shortened dolichol‐linked oligosaccharides Man_5_GlcNAc_2_ and Man_9_GlcNAc_2_. B, Thin‐layer chromatography (TLC) analysis of hydrophobic LLO extracts further revealed that dolichol‐phosphate‐mannose (Dol‐P‐Man) is synthesized in patient 1 fibroblasts. C, Analysis of O‐mannosylated αDG in P1 and control fibroblasts. IIH6 and laminin (laminin overlay, LO) only bind to fully functional O‐mannosyl glycans of αDG. DAG1 binds to the core of the dystroglycan protein, showing expression of αDG and βDG proteins. CDG, congenital disorders of glycosylation; HPLC, High‐performance liquid chromatography; LLO, lipid linked oligosaccharide; MPDU1, mannose‐phosphate‐dolichol utilization defect 1

Next, we analyzed the LLO composition in fibroblasts from patient 1. Cells were incubated with [2‐^3^H]mannose and [^3^H]oligosaccharides were extracted and analyzed using HPLC. Increased levels of dolichol‐linked Man_5_GlcNAc_2_ and Man_9_GlcNAc_2_ accompanied by reduced amounts of Glc_3_Man_9_GlcNAc_2_ were detected (Figure 2A) which indicated shortage of DPM and DPG in the ER lumen. Since TLC analysis further showed that DPM is synthesized in the patient's fibroblasts (Figure 2B), a lack of transport of DPM from the cytosol into the ER can be assumed.

Subsequently, we analyzed the O‐mannosylation of αDG in fibroblasts of patient P1 by IIH6 immunolabeling and a LO assay. We found that the signals for both IIH6 and LO were reduced in patient fibroblasts as compared to the signal of control fibroblasts (Figure 2C), suggesting reduced glycosylation of αDG.

## 4 DISCUSSION

Here, we describe two patients with mutations in *MPDU1*, causing MPDU1‐CDG (CDG‐1f) with overlapping symptoms of CDG‐I and dystroglycanopathy. MPDU1‐CDG is the fifth disorder related to DPM biosynthesis or utilization that bridges CDG‐I and the O‐mannosylation disorders.

So far, seven MPDU1‐CDG patients have been described. All patients showed psychomotor retardation and most patients had hypotonia, facial dysmorphism, eye defects, apnea, and skin abnormalities such as ichthyosis.^7^, ^8^, ^9^ Including the two patients described here, four patients have been described with the same G73E substitution.^7^, ^9^ Hypertelorism, dysphagia, small renal cysts, thrombocytopenia, cardiomyopathy, and respiratory problems characterized these patients, whereas these symptoms were not reported in the other three MPDU1‐CDG patients. In addition, all G73E patients died at an early age (<11 months), whereas the other MPDU1‐CDG patients at least reached their teenage years. Taken together, this suggests that the G73E substitution affects MPDU1 function more severely, and the additional clinical features can aid in the prediction of the disease progression when new MPDU1‐CDG patients are identified.

Interestingly, the two siblings described here showed a very similar clinical pattern, including very similar facial features, whereas siblings with other CDG disorders do not necessarily share as many clinical characteristics, for example, in ALG3‐CDG.^16^ The MPDU1‐CDG siblings shared the following abnormalities: massive dilatation of the intrahepatic biliary duct system, small renal cysts, buphthalmos with glaucoma, DCM, thrombocytopenia, elevated CK, and low ATIII. MPDU1 has been associated with the flipping of DPM over the ER membrane and thereby is part of the DPM biosynthesis defects.^6^ DPM is required for multiple glycosylation pathways. Thus, different symptoms can be caused by dysfunction of different glycosylation pathways. Buphthalmos, glaucoma, DCM, and elevated CK are clinical features that overlap with the disease spectrum of the dystroglycanopathies. Buphthalmos has so far not been described in any of the other DPM disorders, but is associated with Walker‐Warburg syndrome (WWS) and muscle‐eye‐brain (MEB) disease, which are severe variants of dystroglycanopathy.^17^ Glaucoma is a common feature of WWS and MEB, and has also been reported in SRD5A3‐CDG.^17^, ^18^

Unfortunately, the role of glycosylation in cardiac disease is not completely understood. Therefore, there is no clear explanation why patient 1 shows DCM, whereas patient 2 developed HCM. HCM has been reported in PMM2‐CDG, ATP6V1A‐CDG, and ATP6V1E1‐CDG.^19^ Recent studies have shown that abnormal glycosylation, for example, abnormal sialylation or reduced hybrid/complex N‐glycosylation, is related to heart disease.^19^, ^20^, ^21^ However, future investigations are required to understand the clinical relevance of these findings. So far, DCM has been described in FKRP‐CDG, FKTN‐CDG, POMT1‐CDG, POMT2‐CDG, DOLK‐CDG, DPM3‐CDG, and PGM1‐CDG.^19^, ^22^ With the exception of PGM1‐CDG, these CDGs are associated with abnormal O‐mannosylation of αDG. Hence, the cardiac pathomechanism in MPDU1‐CDG patient 1 could be related to abnormal O‐mannosylation, although studies in heart biopsies are warranted to study this. In line with the clinical symptoms overlapping with dystroglycanopathies, we showed reduced O‐mannosylation of αDG in patient fibroblasts.

In addition, we reported biliary duct abnormalities and renal cysts in our MPDU1‐CDG patients. Biliary duct abnormalities have also been observed in patients with mutations in mannose‐6‐phosphate isomerase (MPI), which interconverts fructose‐6‐phosphate to mannose‐6‐phosphate.^23^ Sabry et al^24^ reported a dehydrodolichyl diphosphate synthase (DHDDS‐CDG) patient with dilations of the biliary duct and renal failure, and Schenk et al^7^ reported renal cysts in a MPDU1‐CDG patient with the same G73E substitution. Biliary duct abnormalities and renal cysts are clinical symptoms associated with Caroli syndrome. In some cases, this syndrome has been associated with *PKHD1* mutations, which are also known to cause autosomal recessive polycystic kidney disease. PKHD1 is extensively glycosylated,^25^ and it is tempting to speculate that abnormal glycosylation of PKHD1 causes the biliary duct abnormalities and renal cysts.

In summary, we reported on two newly identified MPDU1‐CDG patients and showed reduced N‐glycosylation and O‐mannosylation in patient material. Together with the muscular, eye, and heart abnormalities, this adds MPDU1‐CDG to the list of DPM disorders causing biochemical and clinical abnormalities overlapping CDG‐I and dystroglycanopathy. Identification of additional patients with defects in DPM availability, and extensive O‐mannosylation and N‐glycosylation analysis of patient material is required to further increase our understanding of the pathophysiology of the DPM disorders.

## CONFLICT OF INTEREST

The authors declare that they have no conflict of interest.

## AUTHOR CONTRIBUTIONS

W.V.T. has set up the study, performed laboratory experiments, acquired and interpreted data, and written the manuscript. A.A. has performed WES analysis and critically reviewed the results and revised the manuscript. E.K. has collected, analyzed, and reviewed clinical data and written the manuscript. N.A.B. has performed ESI‐MS analysis and critically reviewed the manuscript. M.A.W. has critically reviewed results and revised the manuscript. C.T. has provided LLO analysis and revised the manuscript. D.J.L. contributed to the setup of the study, has interpreted data, critically reviewed the results and the manuscript, and supervised the study.

## PATIENT CONSENT

All procedures followed were in accordance with the Helsinki Declaration of 1975, as revised in 2000. Informed consent was obtained from all patients for being included in this study or from their legal representatives. Written informed consent was obtained for inclusion of facial images.
